# Identification of an immune-related eRNA prognostic signature for clear cell renal cell carcinoma

**DOI:** 10.18632/aging.205479

**Published:** 2024-01-29

**Authors:** Yang Lv, Lili Niu, Qiang Li, Wenchuan Shao, Xinghan Yan, Yang Li, Yulin Yue, Hongqi Chen

**Affiliations:** 1 Department of Urology, The Affiliated Jiangsu Shengze Hospital of Nanjing Medical University, Suzhou 215228, China; 2 Central Laboratory, First Affiliated Hospital, Institute (College) of Integrative Medicine, Dalian Medical University, Dalian 116021, China; 3 Department of Pharmacy, Shanghai Pudong Hospital, Fudan University Pudong Medical Center, Shanghai 201399, China; 4 Department of Urology, The First Affiliated Hospital of Soochow University, Suzhou, China; 5 Department of Urology, The State Key Lab of Reproductive, The First Affiliated Hospital of Nanjing Medical University, Nanjing 210029, China; 6 Department of Urology, Shanghai Pudong Hospital, Fudan University Pudong Medical Center, Shanghai 201399, China; 7 Department of Clinical Laboratory, Children’s Hospital of Nanjing Medical University, Nanjing, China

**Keywords:** non-coding RNA, enhancer RNA, immune signature, tumor microenvironment, clear cell renal cell carcinoma

## Abstract

Background: Immune-related enhancer RNAs (eRNAs) have garnered significant attention in cancer metabolism research, yet their specific roles in ccRCC have remained elusive.

Methods: We retrieved eRNA expression profiles from TCGA database and identified immune-related eRNAs (IREs) by assessing their co-expression with immune genes. Utilizing consensus clustering, we organized these IREs into two distinct clusters. The construction of an IREs signature was accomplished through the LASSO and multivariate Cox analysis. Furthermore, we performed Cell Counting Kit-8 and clonogenic assays to assess changes in the proliferative capacity of Caki-1 and 769-P cells.

Results: The existence of two clusters of immune-related eRNAs in ccRCC, each with distinctive prognostic and immunological attributes. Cluster B exhibited immunosuppressive properties and displayed a positive correlation with immunosuppressive cells. Functional enrichment analysis unveiled their involvement in several tumor-promoting pathways, metabolic pathways and immune pathways. The IREs signature demonstrated its potential to accurately predict patient immune and prognostic characteristics. AC003092.1, an eRNA strongly associated with patient survival, emerged as a potential oncogene significantly linked to adverse prognosis and the presence of immunosuppressive cells and checkpoints in ccRCC patients. Notably, AC003092.1 displayed marked upregulation in ccRCC tissues and cell lines, and its knockdown substantially inhibited the proliferation of Caki-1 and 769-P cells.

Conclusion: We established a robust predictive model that played a vital role in determining the prognosis, clinicopathological characteristics and immune cell infiltration patterns of ccRCC patients. IRE, particularly AC003092.1, which was strongly associated with survival, hold promise as novel immunotherapeutic targets for ccRCC.

## INTRODUCTION

Renal cell carcinoma (RCC) is an intricate malignancy that originates from epithelial cells, with renal clear cell carcinoma (ccRCC) being the most common subtype [1]. The incidence and mortality of ccRCC have been steadily increasing, now accounting for approximately two to three percent of adult malignancies [2, 3]. Given its insensitivity to targeted and immunosuppressive agents, surgical intervention remains the primary and most effective treatment modality [4]. Despite significant advancements in early screening and diagnosis, approximately 30% of patients present with metastases at the time of diagnosis and about 25% develop metastases following surgical treatment [5, 6]. Consequently, the urgency lies in the quest for an effective prognostic signature and potential biomarkers to enhance the treatment of ccRCC patients.

RCC represents a prototypical immunogenic tumor that predominantly relies on inducing immunosuppressive cells, such as regulatory T cells, myeloid-derived suppressor cells, and macrophages, to create an immunosuppressive microenvironment [7]. The immune microenvironment has a dual role—it can inhibit tumor growth but also facilitate tumor progression by altering tumor immunogenicity or immunosuppression status [8]. Within the RCC tumor microenvironment, immunosuppressive cells may disrupt immune surveillance, ultimately leading to tumor immune evasion or escape [9]. RCC achieves this by upregulating the expression of immunosuppressive checkpoints, thereby inhibiting the activity of effector T cells and antigen-presenting cells, thus promoting tumor metastasis [10]. Consequently, it becomes imperative to explore the immune-related prognostic model for ccRCC.

Long noncoding RNAs (lncRNAs) are RNA transcripts exceeding 200 nucleotides in length that do not encode proteins; they are distributed widely in both the cytosol and nucleus [11, 12]. A growing body of evidence underscores the pivotal role of lncRNAs in regulating gene expression, translation and tumor progression [13, 14]. eRNAs are a class of RNA transcribed from enhancer regions on the genome, and they are found abundantly in most human cells and tissues [15, 16]. There is mounting evidence linking eRNA transcriptional levels to enhancer activity, implicating them in gene transcriptional regulation and their close association with tumor proliferation and metastasis [17, 18]. Moreover, eRNAs may contribute to tumor progression by regulating nuclear histone structure or interacting with transcriptional regulators [19, 20]. In human cells, eRNA participate in various signal transduction pathways and influence the construction of immune microenvironment by mediating the activation of target genes, thus underscoring the clinical significance of eRNA-targeted therapy [17]. Although immune-related eRNAs (IREs) play a substantial role in gene transcriptional control, their underlying mechanisms in ccRCC remain elusive.

In this study, we conducted a comprehensive evaluation of the prognostic characteristics of IREs in ccRCC. Notably, we established two distinct clusters of IREs, each with unique prognostic and immune characteristics. Additionally, we developed an IREs prognostic model that effectively predicts the survival rates of ccRCC patients. AC003092.1, a key player in the regulation of the tumor immune microenvironment, holds promise in guiding the development of immunotherapies for ccRCC.

## METHODS

### Clinical data acquisition and extraction

Gene expression profiles and clinical data of ccRCC patients were sourced from The Cancer Genome Atlas (TCGA). The dataset comprised 538 cases of ccRCC tissues and 72 cases of normal tissues. For validation, gene expression data for ccRCC were obtained from ArrayExpress (https://www.ebi.ac.uk/arrayexpress) and the International Cancer Genome Consortium (ICGC) (https://icgc.org). The ArrayExpress dataset has the accession number E-MTAB-1980, including 106 cases with follow-up information. The ICGC dataset is labeled as RECA-EU and encompasses 91 cases with follow-up data.

### Identification of immune-related eRNAs

To identify immune-related eRNAs (IREs), we gathered immune-related genes from the Molecular Signatures Database (MSigDB) categories IMMUNE_RESPONSE and IMMUNE_SYSTEM_PROCESS. We determined eRNAs transcribed from active tissue-specific enhancers and predicted their target genes using the Predicted Gene and enhancer Specific Tissue Interaction (PresSTIGE) method [21, 22]. The Pearson correlation analysis was employed to screen for IREs, with the criterion being a |Pearson correlation coefficient| >0.4 and *p* < 0.001. AC003092.1 expression in ccRCC was assessed using quantitative real-time PCR (qRT-PCR). The primers for AC003092.1 were as follows: Forward: TTAGCAGCAAACCCAGAAC; Reverse: TGCTGAGGATACATGACGAA. The primers for GAPDH were: Forward: GAGGTGATAGCATTGCTTTCG; Reverse: CAAGTCAGTGTACAGGTAAGC.

### Construction of immune-related eRNAs clusters and bioinformatics analysis

We conducted univariate Cox regression analysis to identify IREs associated with prognosis. To investigate the function of IREs in ccRCC, we employed the “Consensus ClusterPlus” package for patient classification. Kaplan-Meier (KM) survival curves were generated to assess survival differences among these clusters. To further analyze biological pathways, we screened differential expression genes (DEGs) based on the criteria |log 2 (fold change FC)| >2 and adjusted *P*-value < 0.001. We used Gene Ontology (GO) and Kyoto Encyclopedia of Genes and Genomes (KEGG) for the analysis of molecular roles and associated biological processes of DEGs. Additionally, Gene Set Variation Analysis (GSVA) enrichment analyses were conducted to evaluate pathway enrichment using the R package “GSVA” and “c2.cp.kegg.v7.4.symbols” from the MSigDB. The gene set “c2.cp.kegg.v7.4. symbols” is a commonly used gene set from the MSigDB database, which aggregates information related to gene pathways.

### Analysis of immune characters in immune-related eRNAs clusters

We employed single-sample Gene Set Enrichment Analysis (ssGSEA) to calculate the scores of immune-infiltrating cells and immune-related pathways in individual samples. The “ESTIMATE” R package was used to estimate tumor purity, stromal and immune scores in the tumor microenvironment of ccRCC samples.

### Establishment of immune-related eRNAs signature

Using the expression profile of prognostic IREs, we utilized the Least absolute shrinkage and selection operator (LASSO) regression analysis and multivariate Cox regression analysis to screen key genes and construct the IREs prognostic model. The following formula was used to calculate the risk score for each sample:


$$\text{Riskscore}=\sum_{l}^{i}\left(Coefi\times Gen\;\exp i\right)$$

Here, “Coef” represents non-zero regression coefficients determined through multivariate Cox regression analysis, and “Genexp” is the expression values of genes from IREs prognostic model. Patients were divided equally into high - and low-risk groups according to the median riskscore. KM survival curves were constructed to compare survival differences between these groups. Univariate and multivariate Cox regression analyses were conducted to assess the independence of riskscore and various clinicopathological features including age, gender, histological grade, pathological stage, and TMN stage.

### Prognostic features of immune-related eRNAs signature

To analyze the correlation between riskscore and clinicopathological variables, we further assessed differences in the riskscore among various clinicopathological variables. Chi-square tests were employed to evaluate differences in the distribution of clinicopathological variables between the high - and low-risk groups. Additionally, KM survival curves were used to analyze differences in survival between the high - and low-risk groups within different clinical phenotypes.

### Identification of the key eRNA in ccRCC

Based on the significant IREs expression profiles identified through univariate Cox analysis, patients were divided into high and low expression groups, and survival characteristics in ccRCC were analyzed further. Co-expression analysis was used to assess the correlation between IREs expression levels and their predicted target genes. IREs were included if they demonstrated a significant association with overall survival (OS) (KM log rank *p* < 0.05) and a significant association with predicted target genes (|r| >0.4 and *p* < 0.001). Key IREs, most relevant for survival according to log rank *p*-values, were selected for further analysis. The prognostic features of key IREs and their correlation with clinicopathological features were also investigated.

### Cell culture and plasmid construction

We obtained two human ccRCC cell lines (Caki-1 and 769-P) and a human renal proximal tubular epithelial HK2 cell line (HRPTEpiC) that were purchased from the cell bank of the Chinese Academy of Sciences (Shanghai, China). All cells were cultured in RPMI 1640 medium (Thermo Fisher Scientific, Inc., Waltham, MA, USA) supplemented with 10% fetal bovine serum (FBS; Thermo Fisher Scientific, Inc.) at a constant temperature of 37°C in a humidified atmosphere containing 5% CO_2_.

To silence AC003092.1, two siRNAs were transfected into Caki-1 and 769-P cells using Lipofectamine 3000 (Thermo Fisher Scientific, Inc.), following the manufacturer’s instructions. The sequences used for siRNA-1 were: Sense: GUAAUCCAGCGAAUCUGGA; Antisense: UCCAGAUUCGCUGGAUUAC; siRNA-2: Sense: CAGCAAUCAACAUAAUCAA; Antisense: UUGAUUAUGUUGAUUGCUG.

### Cell counting kit-8 (CCK8) assay

Briefly, Caki-1 and 769-P cells, after various interventions, were incubated in 96-well plates (2 × 10^3^) with 200 μL of culture medium at 37°C with 5% CO_2_. On days one, two, three, four and five, 20 μL CCK-8 solution was added into each well, and incubation was carried out for two hours. Absorbance was measured at an optical density of 450 nm using a Microplate reader (Bio-Rad Laboratories Inc., Hercules, CA, USA). Experiments were conducted in triplicate.

### Cell growth assay and clonal formation assay

For the Cell Growth Assay, cell viability was assessed in accordance with the manufacturer’s instructions. Cells were initially seeded at a density of 2 × 10^3^ cells per well in 96-well plates and evaluated at 0, 24, 48, 72, and 96 hours by the Cell Counting Kit-8 (Beyotime, Shanghai, China) and the Synergy H1 microplate reader (BioTek, Winooski, VT, USA) at 450 nm. In the case of the Clonal Formation Assay, after transfection and selection, 200 cells were distributed in 6-well plates in triplicate and incubated for 14 days. Subsequently, the cells were fixed with 10% ice-cold methanol and stained with 0.5% crystal violet solution. Colonies consisting more than 50 cells per colony were counted, and independent experiments were conducted in triplicate.

### Data availability statement

All data used in this work can be acquired from (TCGA, (https://portal.gdc.cancer.gov/), GEO (https://www.ncbi.nlm.nih.gov/geo/).

## RESULTS

### Establishment of immune-related eRNAs clusters

**Figure 1:**
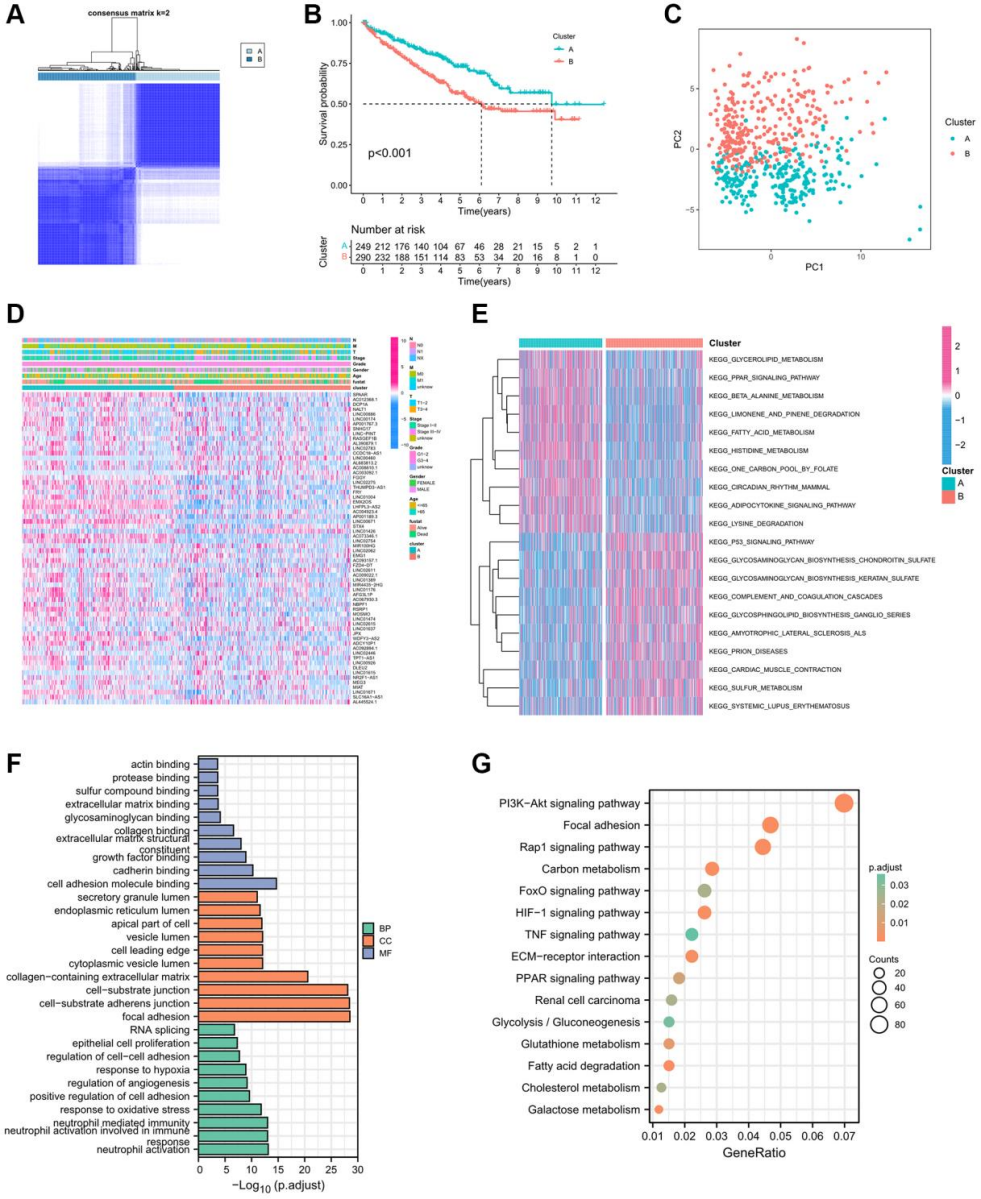
**Establishment biological analysis of immune-related eRNAs clusters.** (**A**) Sample distribution of 2 immune-related eRNAs clusters; (**B**) KM curve showing the survival differences between clusters; (**C**) PCA showing the trend of sample distribution between 2 clusters. (**D**) The heatmap presenting the distribution of immune-related eRNAs and clinical phenotype; (**E**) The heatmap showing the results of GSVA enrichment analysis between 2 clusters. Pink represented activated pathways; blue represented inhibited pathways. (**F**) GO analysis of differential genes between clusters. (**G**) KEGG analysis of intersection genes of differential genes between clusters.

The Supplementary Figure 1 displayed the entire process of this study. Initially, we employed univariate Cox regression analysis on a set of 146 IREs, meticulously selecting 64 IREs linked to prognosis based on the stringent criterion of *P* < 0.01 (Supplementary Table 1). Subsequently, utilizing the expression profile of these prognostic IREs, we categorized patients into two distinct clusters, denoted as cluster A and cluster B, employing the Consensus ClusterPlus package (Figure 1A). KM survival curve analysis revealed a notable difference in survival between these clusters, with cluster B associated with a less favorable prognosis (Figure 1B). Furthermore, principal component analysis (PCA) illustrates the distinction between cluster A and cluster B (Figure 1C). The heatmap was generated to display the distribution of the IREs’ expression and clinicopathological variables, highlighting the higher expression of IREs in cluster A (Figure 1D). Additionally, we employed GSVA to assess differences in biological pathways between the two clusters. The results indicated enrichment of multiple metabolic pathways, including fatty acid metabolism, glycerolipid metabolism and beta alanine metabolism in cluster A, while cluster B exhibited enrichment in multiple pro-cancer pathways, such as the P53 signaling pathway (Figure 1E).

Furthermore, we screened 2619 DEGs for further analysis based on the criteria of |log 2 (fold change FC)| >2 and adjusted *P*-value < 0.001. These DEGs were categorized into Biological Process (BP), Cellular Component (CC), and Molecular Function (MF) groups. In the BP group, genes were primarily enriched in processes related to neutrophil activation, response to oxidative stress, and regulation of angiogenesis. In the CC group, genes were concentrated in focal adhesion, cell–substrate adherens junctions, and cell–substrate junctions. In the MF group, genes were significantly enriched in functions related to cell adhesion molecule binding, cadherin binding, and growth factor binding (Figure 1F). The KEGG enrichment analysis highlighted the significant enrichment of DEGs in multiple pro-cancer pathways, renal cell carcinoma, metabolic pathways and hypoxia related pathways (Figure 1G).

### Identification immune characteristics of immune-related eRNAs clusters

**Figure 2:**
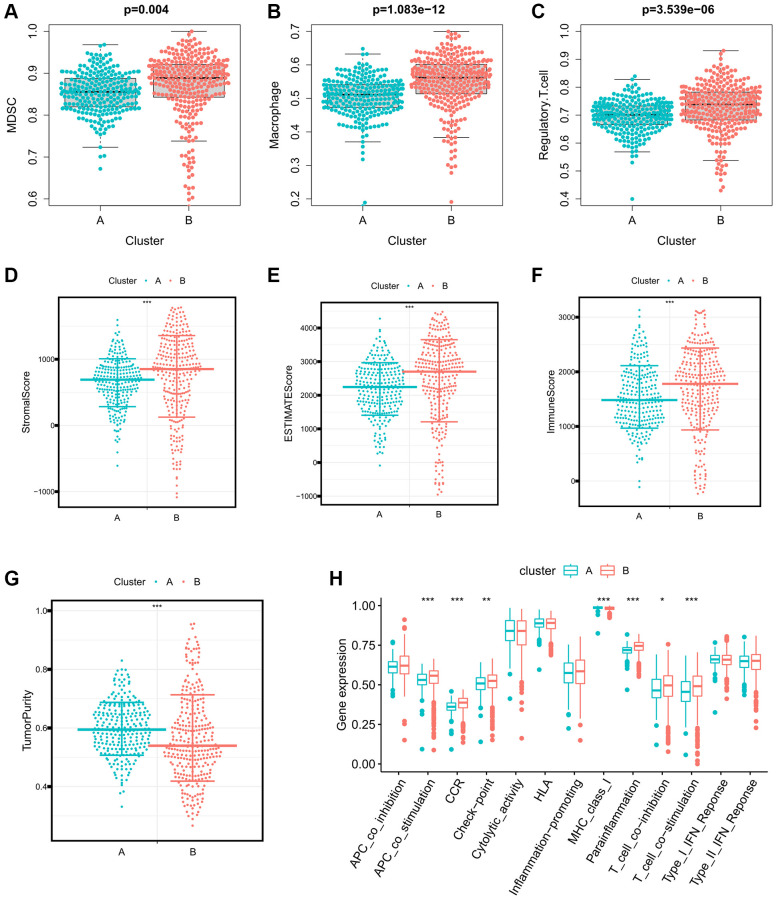
**Differences in immune characteristics between immune-related eRNAs clusters.** (**A**–**C**) Differences in expression of immunosuppressive cells between 2 clusters (**A**: MDSC; **B**: Macrophage; **C**: Regulatory.T.cell); (**D**–**G**) Differences in the expression of tumor microenvironment score between 2 clusters (**A**: StromalScore; **B**: ESTIMATEScore; **C**: ImmuneScore; **D**: TumorPurity); (**H**) Differences in expression of immune function pathways between 2 clusters.

To investigate the immune characteristics of the Immune-Related eRNAs clusters, we utilized the ssGSEA algorithm to calculate the immunosuppressive cell infiltration score in individual ccRCC samples. Notably, immunosuppressive cells, including Myeloid-Derived Suppressor Cells (MDSCs), Macrophages, and Regulatory T cells, exhibited significant overexpression in cluster B (Figure 2A–2C). Furthermore, we applied the ESTIMATE algorithm to calculate the estimated score, immune score, stromal score and tumor purity of the tumor microenvironment. Our findings indicated that estimated score, immune score, and stromal score were significantly higher in cluster B, while tumor purity was significantly lower in cluster B (Figure 2D–2G). We further analyzed the differential expression of immune function pathways among the different clusters, revealing that pathways such as Antigen-Presenting Cell (APC) co-stimulation, Cytokine Receptor Regulation (CCR), Checkpoint Pathways, and Parainflammation were significant higher in the cluster B (Figure 2H).

### Development of immune-related eRNAs signature

**Figure 3:**
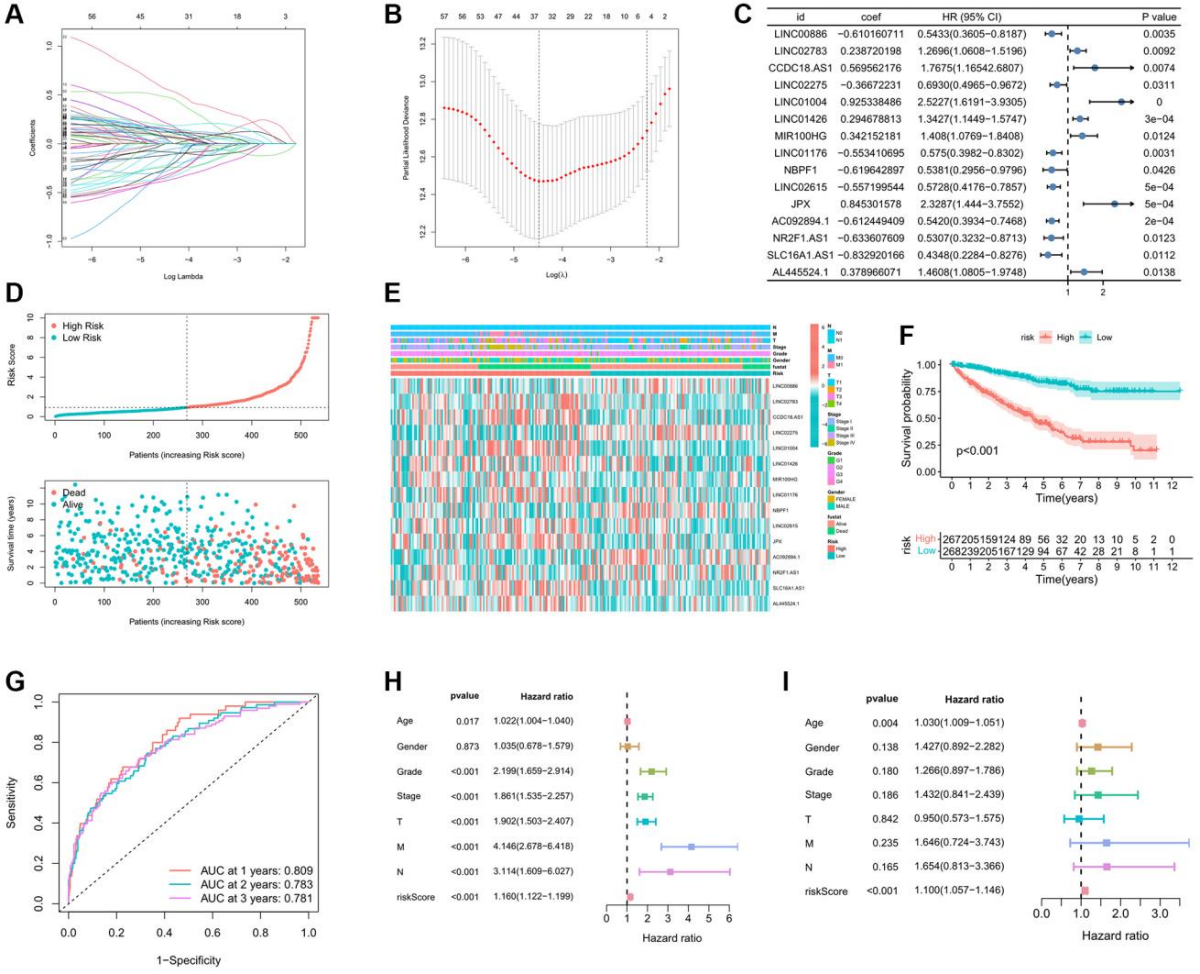
**Establishment prognostic analysis of immune-related eRNAs signature.** (**A**) LASSO coefficient profiles of the expression of prognostic immune-related eRNAs. (**B**) Selection of the penalty parameter (λ) in the LASSO model via 10-fold cross-validation. (**C**) Results of immune-related eRNAs multivariate analysis; (**D**) Relationship between the survival status/immune-related eRNAs signature rank and survival time (years)/immune-related eRNAs signature rank; (**E**) Distribution of immune-related eRNAs expression and clinical phenotype between high and low risk groups; (**F**) KM curve showing the survival differences between high and low risk groups; (**G**) Time-dependent ROC curve for OS of the riskscore. The AUC was assessed at 1, 2 and 3 years; The univariate (**H**) and multivariate (**I**) Cox regression analysis of riskscore, age, gender, grade, stage, and TMN.

In the process of developing the IREs signature, we initially employed Lasso regression analysis to further refine our selection from the initial 64 prognostic IREs, ultimately identifying 33 prognostic IREs (Figure 3A, 3B). Subsequently, we utilized multivariate Cox regression analysis and identified the 15 most relevant IREs with the lowest Akaike information criterion (AIC) value, which were then used to construct the IREs signature (Figure 3C). Based on the median of riskscore, ccRCC patients were equally divided into high and low risk groups. The riskscore exhibited an inverse association with patient survival in the ccRCC sample, clearly depicted in Figure 3D. Furthermore, the heatmap illustrated the distribution of the modeled genes and clinicopathological variables (Figure 3E). The KM survival curve analysis highlighted that patients in the high-risk group had a significantly worse prognosis compared to those in the low-risk group (Figure 3F). We assessed the predictive accuracy of the riskscore by calculating the Area Under the Curve (AUC) for one-year, two-year, and tree-year risk scores, resulting in AUC values of 0.809, 0.783, and 0.781, respectively. This demonstrated that the riskscore was effective in accurately predicting patient prognosis (Figure 3G). To further validate the independence and accuracy of the riskscore in predicting patient prognosis, we performed univariate and multivariate independent prognostic analyses, considering the riskscore and clinicopathological variables. The univariate independent prognostic analysis demonstrated that age, grade, stage, TMN stage, and riskscore significantly impacted OS (Figure 3H). Meanwhile, the multivariate independent prognostic analysis confirmed that the riskscore remained an independent prognostic indicator for OS (HR: 1.100, 95%CI: 1.057−1.146, *p*-value < 0.001) (Figure 3I).

### Identification of clinical characteristic of immune-related eRNAs signature

**Figure 4:**
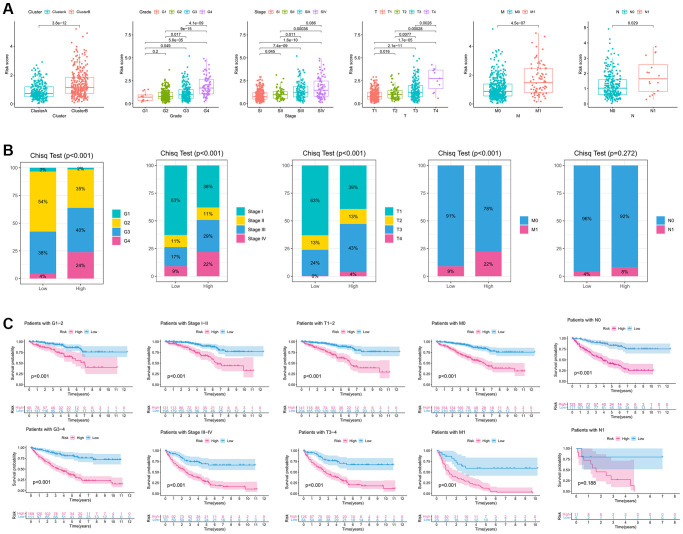
**Clinicopathological characteristic of the immune-related eRNAs signature.** (**A**). Differences in the expression of riskscore in various clinicopathological stages; (**B**) Differences in the proportion of different clinicopathological stages between high and low riskscore groups. (**C**) KM survival curve showing the survival differences between high and low score groups in different clinicopathological stages.

To further investigate the relationship between riskscore and clinicopathological variables, we initially examined the differences in riskscore expression among various clinicopathological variables. Interestingly, significant variations in riskscore expression were observed among different IREs clusters, histological grade, pathological stage, and TNM stage (Figure 4A). Notably, a trend was observed where more advanced clinical phenotypes were associated with higher riskscores. Additionally, we conducted chi-square tests to evaluate differences in the distribution of clinicopathological variables between high and low-risk groups. The results revealed that the high-risk group had a higher proportion of advanced clinicopathological variables, and these differences were statistically significant, except for the N stage (Figure 4B). Furthermore, through an analysis of the differences in survival between the high and low risk groups within various clinical phenotypes, we observed that the high-risk group consistently exhibited a poorer prognosis across all clinical phenotypes, with statistically significant differences (Figure 4C).

### Identification of prognostic characteristics of the key eRNA---AC003092.1

**Figure 5:**
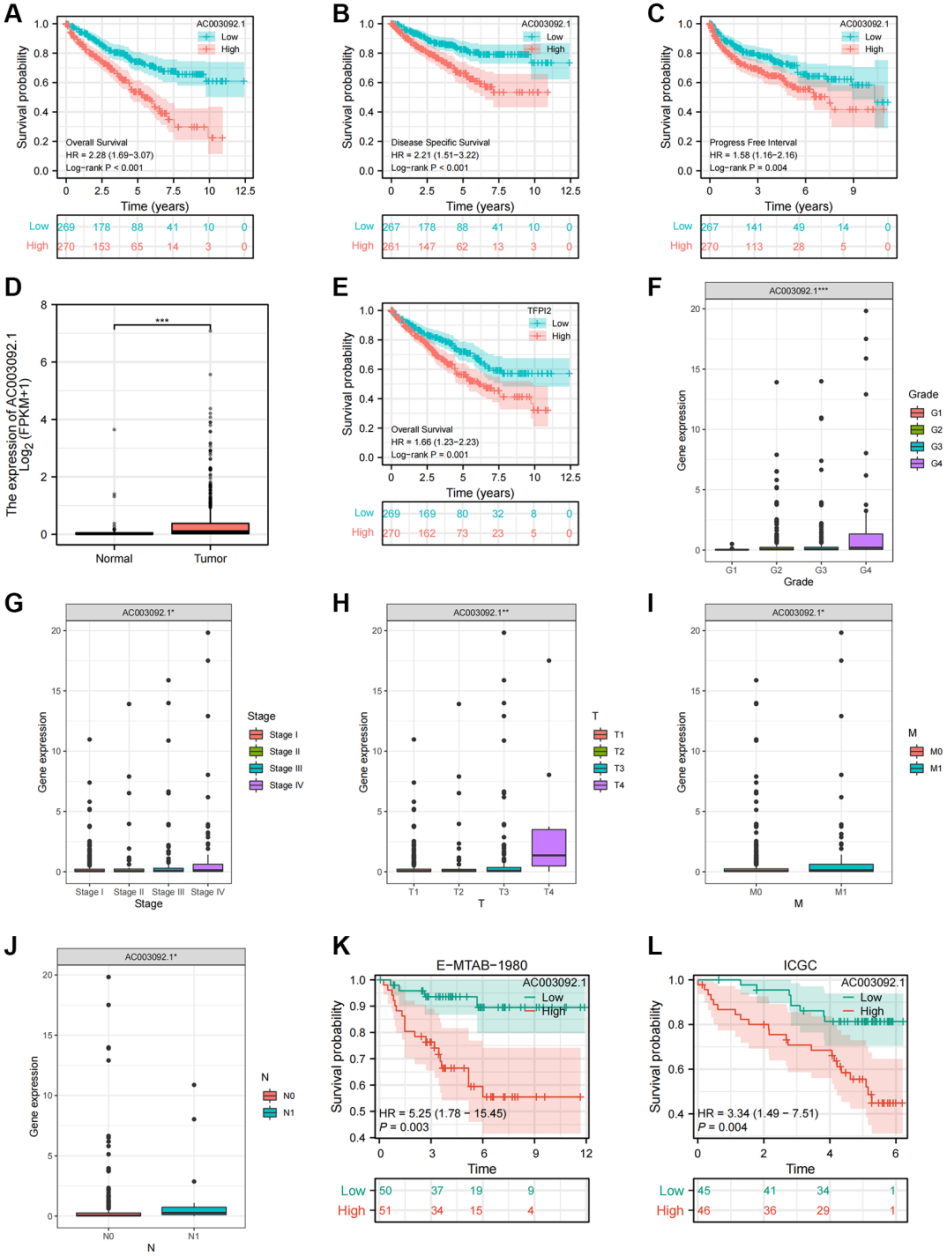
**Clinical and prognostic characteristics of AC003092.1 in ccRCC.** (**A**–**C**) KM survival curve showing the survival differences between high and low AC003092.1 groups (**A**: OS; **B**: DSS; **C**: PFI); (**D**) Difference of AC003092.1 mRNA expression between ccRCC and adjacent tissues; (**E**) KM survival curve showing the survival differences between high and low TFPI2 groups; (**F**–**J**) Differences of AC003092.1 expression in different clinicopathological variables (**F**: Grade; **G**: Stage; **H**: T stage; **I**: M stage; **J**: N stage); (**K**, **L**) KM survival curve showing the survival differences between high and low AC003092.1 groups in the E-MTAB-1980 and ICGC datasets.

As detailed in Supplementary Table 2, AC003092.1 emerged as the eRNA most strongly associated with survival, with a notably positive correlation to its predicted target, TFPI2. Subsequently, patients were evenly stratified into high and low AC003092.1 expression groups based on the median expression of AC003092.1 in ccRCC. Differences in survival outcomes, including OS, disease specific survival (DSS), and progress free interval (PFI) were assessed between these two groups. The analysis revealed that the high AC003092.1 group exhibited an adverse prognosis in OS, DSS, and PFI compared to the low AC003092.1 group, and these differences held statistical significance (Figure 5A–5C). Furthermore, when comparing AC003092.1 expression levels between ccRCC and adjacent tissues, AC003092.1 was found to be significantly upregulated in ccRCC (Figure 5D). The KM survival curve demonstrated that the high TFPI2 group was associated with a less favorable prognosis for ccRCC patients (Figure 5E). Additionally, we delved into the relationship between AC003092.1 expression and clinicopathological characteristics in ccRCC, revealing a significant positive correlation between the expression level of AC003092.1 and several clinicopathological features, including patient status, histological grade, pathological stage, and TNM stage (Figure 5F–5J). Furthermore, external validation sets, E-MTAB-1980 and ICGC, corroborated the association of AC003092.1 expression with a poor prognosis (Figure 5K, 5L).

### Identification of immune characteristics of the key eRNA---AC003092.1

**Figure 6:**
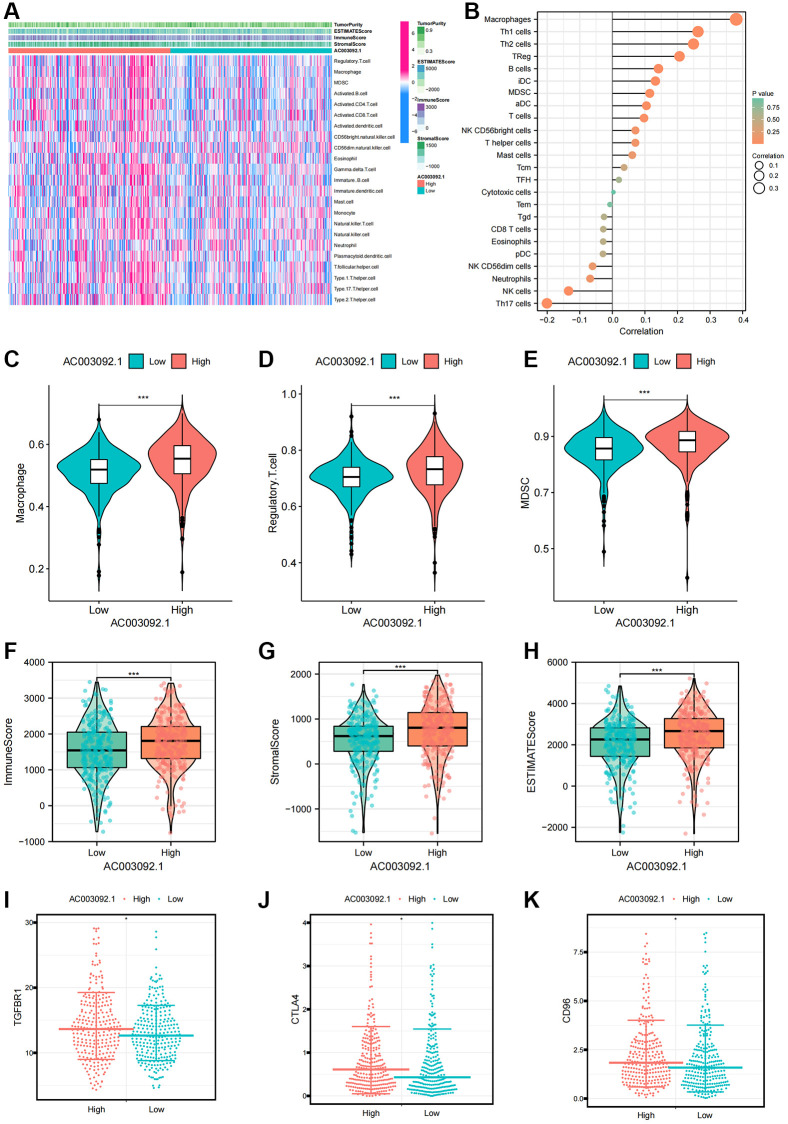
**Immune characteristics of AC003092.1 in ccRCC.** (**A**) Distribution of immune infiltrating cells and tumor microenvironment scores in high and low AC003092.1 expression groups; (**B**) Correlation between immunoinfiltrating cells and AC003092.1 expression profiles. (**C**–**E**) Differences in immunosuppressive cell expression between high and low AC003092.1 groups (**C**: Macrophage; **D**: Regulatory.T.cell; **E**: MDSC); (**F**–**H**) Differences in tumor microenvironment scores between high and low AC003092.1 groups (**F**: ImmuneScore; **G**: StromalScore; **H**: ESTIMATEScore); (**I**–**K**) Differences in immune suppression checkpoints between high and low AC003092.1 groups (**I**: TGFBR1; **J**: CTLA4; **K**: CD96).

To gain deeper insights into the role of AC003092.1 in the tumor microenvironment, we explored the correlation between AC003092.1 and immune cells as well as tumor microenvironment scores. A heatmap highlighted that immunoinfiltrating cells and tumor microenvironment score were notably upregulated in the high AC003092.1 group (Figure 6A). Figure 6B presented the correlation between AC003092.1 and immune infiltrating cells, revealing a significantly positively correlation with immunosuppressive cells (MDSC, Macrophage and Regulatory.T.cell) and a negative correlation with NK cells and neutrophils. This analysis was complemented by a noticeable increase in the expression of immunosuppressive cells in the high AC003092.1 group when compared to the low AC003092.1 group (Figure 6C–6E). Additionally, the tumor microenvironment score, encompassing estimated score, immune score, and stromal score, was significantly elevated in the high AC003092.1 group (Figure 6F–6H). Further exploration of the correlation between AC003092.1 and immunosuppressive checkpoints revealed that immunosuppressive checkpoints (TGFBR1, CTLA4, and CD96) were also significantly upregulated in the high AC003092.1 group (Figure 6I–6K).

### AC003092-knockdown suppressed proliferation in Caki-1 and 769-P cells

**Figure 7:**
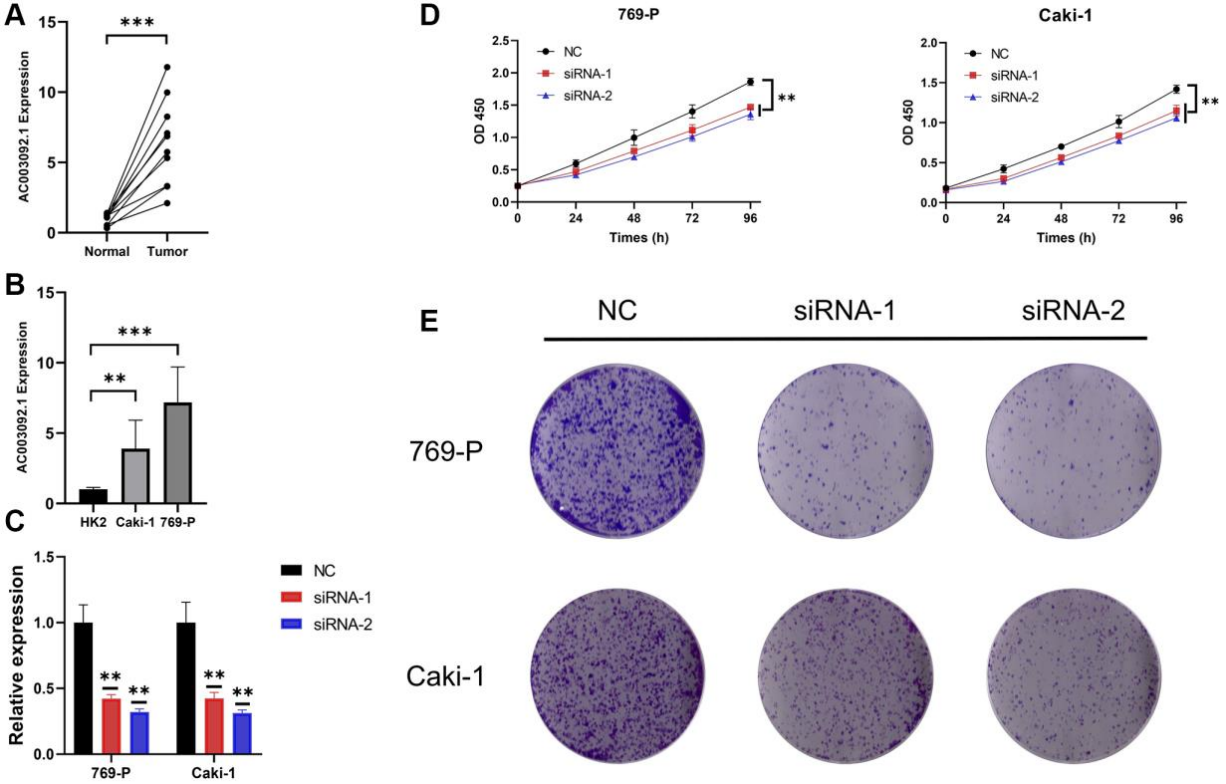
**AC003092-knockdown suppressed proliferation in caki-1 and 769-P cells.** (**A**) Differences in AC003092.1 expression between ccRCC tissues and adjacent tissues; (**B**) Differences in AC003092.1 expression between HK2 and renal cell lines 769-P and Caki-1; (**C**) The expression of AC003092 was downregulated in 769-P and Caki-1 cells, respectively, as determined by RT-qPCR; (**D**) AC003092-knockdown suppressed ccRCC cell proliferation in 769-P and Caki-1 cells; (**E**) Clonogenic formation in AC003092-knockdown 769-P and Caki-1 cells, along with images of formed clones following control cells for two weeks.

RT-qPCR results indicated that AC003092.1 was substantially upregulated in ccRCC tissues (Figure 7A). Comparative analysis with HK2 cell lines showed a significant increase in AC003092.1 expression in renal cell lines, particularly in 769-P and Caki-1, with the highest expression observed in Caki-1 (Figure 7B). Subsequently, AC003092.1-shRNA was transfected into 769-P and Caki-1 cells to effectively knock down AC003092.1, confirmed by RT-qPCR (Figure 7C). Next, CCK8 assay revealed that AC003092.1 knockdown led to a reduction in proliferation in both 769-P and Caki-1 cells (Figure 7D). In the clonogenic assay, AC003092.1 knockdown resulted in a significantly lower number of colonies formed in both 769-P and Caki-1 cells compared to the empty vector controls (Figure 7E).

## DISCUSSION

Epidemiological studies have revealed a rising incidence and mortality rate of RCC. RCC is characterized as an immunogenic tumor with infiltrating myeloid cell, including macrophages and neutrophils as well as CD8+ T cells and natural killer (NK) cells [23]. Despite the identification of immune cells and immune checkpoints as novel prognostic biomarkers and therapeutic targets for ccRCC, only a fraction of patients with ccRCC benefit from such approaches [9, 24]. The dysregulation of eRNAs is closely linked to various human diseases and immune microenvironment, making eRNAs a promising target for effective therapeutic interventions [25, 26]. Therefore, our objective was to construct a novel IREs prognostic model for ccRCC, aiming to identify reliable predictive and prognostic biomarkers while exploring new immunotherapeutic targets.

eRNA represents a noncoding RNA transcribed by enhancers, facilitating the activation of target genes [27, 28]. These eRNAs not only play a role in regulating the immune response but are also involved in numerous tumorigenic signaling pathways, such as p53 and immune checkpoints, which hold pivotal roles in tumor progression and metastasis [29]. For instance, KLK3 eRNA (KLK3e) selectively enhances the expression of androgen receptor-regulated genes, thereby promoting the proliferation and metastasis of prostate cancer [30]. NET1e is significantly overexpressed in breast cancer and is associated with poor prognosis [17]. As our understanding of eRNA mechanisms deepens, studies have revealed that eRNAs fulfill diverse biological roles in the metastasis and progression of various tumors [31]. Fan et al. constructed an eRNA-related prognostic model in prostate cancer that effectively predicted patient outcomes and explored the immune characteristics of this model [32]. The similarity between our study and other model-based research lies in the fact that both involve clustering key genes, building models, and subsequently analyzing the model’s immune, mutation, and prognostic features [33–38]. Notably, our study extended beyond the prognosis and immune characteristics of the IREs signature; we also identified the most relevant eRNA for survival and conducted in-depth analyses of its immune and clinicopathological characteristics.

In our investigation, AC003092.1 emerged as the IRE most strongly associated with survival in ccRCC. AC003092.1 was significantly upregulated in ccRCC and exhibited a close association with poor prognosis and clinicopathological staging. Furthermore, AC003092.1 displayed a significant positive correlation with immunosuppressive cells and immunosuppressive checkpoints, suggesting its potential involvement in shaping an immunosuppressive microenvironment. Notably, Guo et al. demonstrated that AC003092.1 was an IRE, linked to immune cell composition, function, and pathways, possibly contributing to the formation of glioblastoma multiforme (GBM). In GBM patients, AC003092.1 was significantly correlated with poor prognosis and the upregulated expression of its target gene, TFPI2 [39]. AC003092.1’s role in GBM involves enhancing the sensitivity of GBM to temozolomide through the mediation of the miR-195/TFPI-2 signaling pathway, impacting patient prognosis. Furthermore, AC003092.1 can counteract the upregulation of TFPI2 expression by miR-195, thus promoting temozolomide-induced apoptosis [40]. These findings highlighted AC003092.1 as a promising molecular target for preventive and therapeutic strategies of ccRCC. However, a comprehensive understanding of the molecular mechanisms governing AC003092.1’s aberrant regulation and its role in ccRCC progression necessitates further investigation, ideally through *in vivo* and *in vitro* experimental analysis.

Nonetheless, it is important to acknowledge certain limitations in our study. Firstly, our data analysis relied on publicly available datasets, and additional datasets should be employed for further validation of our results. Additionally, the biological mechanism underlying AC003092.1’s influence on reshaping the immunosuppressive microenvironment warrant exploration through in-depth *in vivo* and *in vitro* experiments.

## CONCLUSION

IREs played a pivotal role in shaping the immunosuppressive TME in ccRCC. The IREs signature demonstrated remarkable accuracy in distinguishing the immune characteristics and predicting the prognosis of ccRCC patients. AC003092.1, specifically, exhibited an immunosuppressive effect within the TME and hold promise as a potential therapeutic target for ccRCC treatment.

## Supplementary Materials

Supplementary Figure 1

Supplementary Table 1

Supplementary Table 2
