# Supplementary figures and images for: Identification of an immune-related eRNA prognostic signature for clear cell renal cell carcinoma
Source: Aging (Albany NY). 2024 Jan 29;16(3):2232–48. doi: 10.18632/aging.205479 (PMC10911372; doi:10.18632/aging.205479)

SUPPLEMENTARY FIGURE

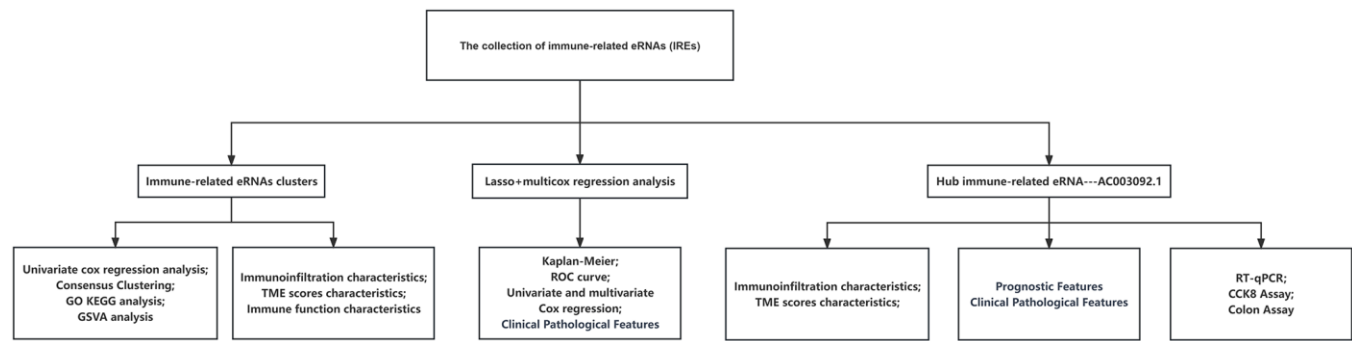

Supplementary Figure 1. The flow chart of the research.

Supplement: Supplementary Figure 1 [file aging-16-205479-s001.pdf]
