# Supplementary material for: Identification of an immune-related eRNA prognostic signature for clear cell renal cell carcinoma
Source: Aging (Albany NY). 2024 Jan 29;16(3):2232–48. doi: 10.18632/aging.205479 (PMC10911372; doi:10.18632/aging.205479)
Supplement: Supplementary Table 1 [file aging-16-205479-s002.docx]

**Supplementary Table 1. Univariate Cox regression analysis results of IREs.**

| id | HR | HR.95L | HR.95H | pvalue |
| --- | --- | --- | --- | --- |
| SNHG17 | 2.254561957 | 1.800568463 | 2.823024905 | 1.38E-12 |
| EMX2OS | 0.647831695 | 0.573796815 | 0.73141902 | 2.36E-12 |
| LINC00460 | 1.558665932 | 1.370473861 | 1.772700345 | 1.38E-11 |
| LINC01615 | 1.731670904 | 1.468528543 | 2.041965159 | 6.61E-11 |
| LINC01004 | 1.769699316 | 1.463443364 | 2.140045695 | 3.92E-09 |
| MIAT | 1.605938223 | 1.370058359 | 1.882428992 | 5.08E-09 |
| NALT1 | 2.067182531 | 1.618966326 | 2.639488881 | 5.76E-09 |
| AC008610.1 | 1.678321875 | 1.406344404 | 2.002897946 | 9.46E-09 |
| LINC00926 | 2.474440936 | 1.799205999 | 3.40308889 | 2.51E-08 |
| LINC01671 | 0.73843465 | 0.661768793 | 0.82398224 | 5.90E-08 |
| AL390879.1 | 2.360805911 | 1.728835639 | 3.223790871 | 6.52E-08 |
| AL445524.1 | 1.826030857 | 1.456827859 | 2.288800746 | 1.74E-07 |
| LINC01389 | 2.121155714 | 1.578814455 | 2.849797547 | 6.00E-07 |
| AC003092.1 | 1.345396267 | 1.196350476 | 1.513010738 | 7.32E-07 |
| LINC02446 | 1.658437384 | 1.353414127 | 2.032204705 | 1.07E-06 |
| AFG3L1P | 2.065846923 | 1.534576839 | 2.781042565 | 1.72E-06 |
| AC009022.1 | 2.05593536 | 1.529358873 | 2.763818406 | 1.80E-06 |
| STX4 | 2.308637401 | 1.637278647 | 3.255283797 | 1.82E-06 |
| CCDC18-AS1 | 1.60321579 | 1.319642813 | 1.947724674 | 2.01E-06 |
| LINC00174 | 1.672904024 | 1.352921075 | 2.068566987 | 2.03E-06 |
| WDFY3-AS2 | 0.516247513 | 0.392926048 | 0.678273929 | 2.06E-06 |
| AC092894.1 | 0.484891724 | 0.35898691 | 0.654954199 | 2.37E-06 |
| LINC02754 | 0.508759287 | 0.383554899 | 0.674834327 | 2.75E-06 |
| FRY | 0.525277392 | 0.40102297 | 0.688031257 | 2.94E-06 |
| SLC16A1-AS1 | 1.929553958 | 1.461939319 | 2.546739409 | 3.45E-06 |
| SPAAR | 0.622231123 | 0.507300693 | 0.763199373 | 5.27E-06 |
| LINC02783 | 1.348941907 | 1.183321041 | 1.537743525 | 7.52E-06 |
| LINC02611 | 2.010126557 | 1.477922879 | 2.733978095 | 8.61E-06 |
| MEG3 | 1.69055407 | 1.335303378 | 2.140317407 | 1.29E-05 |
| NBPF1 | 0.382534519 | 0.247178538 | 0.59201199 | 1.61E-05 |
| THUMPD3-AS1 | 1.872493096 | 1.394952284 | 2.513512782 | 2.97E-05 |
| LINC02062 | 1.89449098 | 1.393984536 | 2.574702931 | 4.46E-05 |
| AC093157.1 | 2.340440927 | 1.555147153 | 3.522280011 | 4.56E-05 |
| EMG1 | 1.902304879 | 1.381952422 | 2.618587873 | 8.01E-05 |
| JPX | 1.996973123 | 1.414360829 | 2.819578691 | 8.50E-05 |
| AP001767.3 | 1.952483111 | 1.397233596 | 2.728384366 | 8.88E-05 |
| LINC-PINT | 1.405600633 | 1.183779074 | 1.66898806 | 0.000102234 |
| AP001189.3 | 0.715091968 | 0.600811671 | 0.851109502 | 0.000160292 |
| LINC02275 | 0.563694703 | 0.418310782 | 0.759606809 | 0.00016548 |
| FGGY | 0.619381005 | 0.48259994 | 0.794929294 | 0.000168157 |
| AC073346.1 | 0.709769521 | 0.590568464 | 0.853030263 | 0.00025764 |
| LINC01474 | 1.790234397 | 1.297099914 | 2.470849902 | 0.000396683 |
| RASGEF1B | 0.517632757 | 0.359123444 | 0.74610465 | 0.000415355 |
| AC004923.4 | 1.415826886 | 1.166593928 | 1.718306364 | 0.000432009 |
| LINC01176 | 1.377741002 | 1.147741615 | 1.653830658 | 0.000584681 |
| LINC00886 | 0.519580164 | 0.355407385 | 0.759589019 | 0.000727093 |
| RSRP1 | 1.310569929 | 1.115291672 | 1.540039778 | 0.001018167 |
| AL683813.2 | 1.744684675 | 1.245356752 | 2.444218983 | 0.001214172 |
| LINC01426 | 1.234573262 | 1.085985127 | 1.403491726 | 0.001278831 |
| AC067930.3 | 1.600770669 | 1.202030666 | 2.131781499 | 0.00128673 |
| MIR100HG | 1.428837045 | 1.146408904 | 1.780843898 | 0.001493324 |
| DLEU2 | 1.644727873 | 1.209249133 | 2.237032636 | 0.001520723 |
| DCP1A | 0.552868473 | 0.383032688 | 0.798009043 | 0.001550987 |
| LINC02615 | 1.411344068 | 1.136059473 | 1.753334332 | 0.001856534 |
| FZD4-DT | 0.488353921 | 0.310932752 | 0.767013287 | 0.001861312 |
| LINC01637 | 1.604574085 | 1.18673912 | 2.169523151 | 0.002123477 |
| LINC00671 | 0.867592857 | 0.792058798 | 0.950330163 | 0.002241682 |
| LHFPL3-AS2 | 0.816043864 | 0.716219049 | 0.92978201 | 0.002261391 |
| TPT1-AS1 | 1.65419531 | 1.195604762 | 2.288684531 | 0.002377797 |
| ADCY10P1 | 1.550754104 | 1.164367845 | 2.065359585 | 0.002692747 |
| MIR4435-2HG | 1.459946999 | 1.12023339 | 1.902679619 | 0.005108229 |
| MOSMO | 0.56989092 | 0.382342697 | 0.849436025 | 0.00575732 |
| NR2F1-AS1 | 0.566635379 | 0.377045032 | 0.851557839 | 0.006273924 |
| AC012368.1 | 1.291223505 | 1.067270796 | 1.56216974 | 0.008542339 |
| SNHG26 | 1.604549569 | 1.111545887 | 2.316215057 | 0.011583309 |
| TMEM184A | 1.441809387 | 1.084194938 | 1.91738057 | 0.011877227 |
| LINC00989 | 0.595085399 | 0.396344616 | 0.893481627 | 0.012310393 |
| TULP4 | 0.607047313 | 0.409462385 | 0.899976294 | 0.012972217 |
| LINC01111 | 1.280267181 | 1.051719944 | 1.558479578 | 0.013794245 |
| AL355803.1 | 0.758043653 | 0.607039995 | 0.946610083 | 0.014523158 |
| CTBP1-DT | 0.572551329 | 0.364189152 | 0.90012298 | 0.015700532 |
| MTLN | 1.27342768 | 1.042273467 | 1.55584701 | 0.01802542 |
| FAAHP1 | 1.356859189 | 1.052740685 | 1.748832249 | 0.018427535 |
| CHST12 | 1.959084913 | 1.115412171 | 3.44089279 | 0.019282045 |
| BLCAP | 1.54153276 | 1.065725333 | 2.229770819 | 0.021563893 |
| LINC02048 | 1.286299101 | 1.03724893 | 1.595147827 | 0.021844989 |
| CROCCP2 | 1.297956303 | 1.036092256 | 1.626004397 | 0.023307135 |
| AL590764.1 | 1.49117653 | 1.051183283 | 2.115337524 | 0.025105748 |
| AC073257.2 | 1.279191159 | 1.028761806 | 1.590582011 | 0.026756465 |
| LINC00472 | 0.709642681 | 0.51931905 | 0.96971743 | 0.031319732 |
| HIVEP2 | 0.756455942 | 0.581671286 | 0.983761113 | 0.037333678 |
| TP53TG1 | 1.289386067 | 1.013776639 | 1.639923793 | 0.038314102 |
| AL035587.1 | 1.454601055 | 1.019503308 | 2.075387311 | 0.038783103 |
| AC022400.6 | 1.426257671 | 1.016063448 | 2.0020511 | 0.040163215 |
| DGCR9 | 1.211718594 | 1.002508337 | 1.464588271 | 0.047047754 |
| LINC00205 | 1.320511098 | 0.993525456 | 1.755113118 | 0.055464821 |
| AC116345.1 | 0.855348242 | 0.727570907 | 1.005566066 | 0.058394417 |
| CDK2AP1 | 1.447673452 | 0.984974379 | 2.127728873 | 0.059712523 |
| LCOR | 0.695877213 | 0.468814097 | 1.032914964 | 0.071977728 |
| HSD11B1-AS1 | 1.226885505 | 0.978001124 | 1.53910666 | 0.077116687 |
| IGHA2 | 1.061690685 | 0.992493942 | 1.135711829 | 0.081708496 |
| LINC00987 | 0.739684398 | 0.526407402 | 1.039371801 | 0.082307363 |
| LINC01184 | 0.580318273 | 0.314068784 | 1.072278796 | 0.082354468 |
| HCP5 | 0.847673717 | 0.697300897 | 1.030474409 | 0.097182725 |
| MSN | 0.821347191 | 0.650254574 | 1.037457076 | 0.098655166 |
| STK3 | 0.664819456 | 0.400112318 | 1.104652092 | 0.115076009 |
| TRMT11 | 1.331690943 | 0.91554741 | 1.93698409 | 0.134025019 |
| EDIL3-DT | 1.202757098 | 0.943481146 | 1.533284097 | 0.136141355 |
| LINC02384 | 1.091287791 | 0.972386306 | 1.224728316 | 0.137752967 |
| AC005515.1 | 1.180798486 | 0.945283916 | 1.474990786 | 0.143138097 |
| AC005082.1 | 0.82912967 | 0.643898842 | 1.067645978 | 0.146348546 |
| MIATNB | 1.36263339 | 0.885306425 | 2.09731874 | 0.159637311 |
| CMAHP | 1.161537846 | 0.940679081 | 1.434251272 | 0.164031006 |
| MAL2 | 0.932881364 | 0.842273386 | 1.03323654 | 0.182609661 |
| AL121899.1 | 1.175511139 | 0.916739181 | 1.507327782 | 0.202420122 |
| LINC01150 | 1.197267651 | 0.900176468 | 1.5924098 | 0.215988888 |
| COX10 | 0.752222124 | 0.473663549 | 1.194599257 | 0.227624518 |
| AC091849.2 | 1.200785969 | 0.889332617 | 1.621313462 | 0.232326605 |
| LINC01187 | 0.915278952 | 0.789761519 | 1.060744972 | 0.239456567 |
| MAP4K3-DT | 0.701284239 | 0.385961905 | 1.274217941 | 0.244175527 |
| LINC01094 | 1.15934377 | 0.892371785 | 1.506186097 | 0.268199015 |
| AP004608.1 | 0.881573842 | 0.702461991 | 1.106355147 | 0.276706281 |
| AC090559.1 | 0.881763862 | 0.702033316 | 1.107507992 | 0.279273158 |
| Z94721.1 | 1.14371563 | 0.886748382 | 1.475148385 | 0.301026034 |
| TP73-AS1 | 0.836453177 | 0.572809276 | 1.221443065 | 0.35524316 |
| TMEM9B-AS1 | 1.14767404 | 0.856389298 | 1.538033818 | 0.356477771 |
| AL589843.1 | 0.869970594 | 0.646195508 | 1.171238157 | 0.358546909 |
| IQANK1 | 1.136017208 | 0.849777358 | 1.518674373 | 0.389247828 |
| AL355574.1 | 1.148108026 | 0.837473745 | 1.573962224 | 0.390859725 |
| LINC00513 | 0.855845987 | 0.586409648 | 1.249079644 | 0.419676374 |
| SLC47A1P2 | 0.893159753 | 0.662892577 | 1.203414207 | 0.457626552 |
| TMEM161B-AS1 | 1.117819808 | 0.833085857 | 1.499870767 | 0.457769871 |
| RAB30-DT | 0.870403704 | 0.601842773 | 1.258804858 | 0.46093251 |
| FTX | 0.854035965 | 0.540897809 | 1.348456984 | 0.498361481 |
| AC105206.2 | 1.142148826 | 0.760521287 | 1.715276038 | 0.521792921 |
| C12orf75 | 0.947950674 | 0.802085289 | 1.120342801 | 0.530652538 |
| WAKMAR2 | 1.140182548 | 0.753047682 | 1.726339876 | 0.5353544 |
| LINC00853 | 1.068654637 | 0.849258458 | 1.344729303 | 0.571156524 |
| DARS-AS1 | 1.081631943 | 0.787201591 | 1.486185589 | 0.628356412 |
| AFDN-DT | 0.926568036 | 0.655194349 | 1.310341468 | 0.666223545 |
| LINC01137 | 1.066228808 | 0.795168788 | 1.429688749 | 0.668294424 |
| WARS2-AS1 | 0.898231361 | 0.541686994 | 1.489457174 | 0.677452441 |
| PINLYP | 0.944604231 | 0.719368156 | 1.240362316 | 0.681763324 |
| MIR3936HG | 0.959939079 | 0.780810259 | 1.180162561 | 0.698025299 |
| PARGP1 | 0.928151594 | 0.621380727 | 1.38637287 | 0.715709289 |
| AC116351.1 | 0.970651424 | 0.807210763 | 1.167184868 | 0.751515857 |
| RFX3-AS1 | 1.055875463 | 0.749996072 | 1.48650511 | 0.755392113 |
| MSH6 | 0.9467396 | 0.644641342 | 1.390410157 | 0.78015874 |
| RYK | 0.940688837 | 0.611945432 | 1.446036593 | 0.780465471 |
| TEX41 | 1.021914097 | 0.80689928 | 1.29422401 | 0.857269227 |
| PCBP1-AS1 | 1.037836353 | 0.676805573 | 1.591453054 | 0.864802672 |
| FLVCR1-DT | 1.0271326 | 0.741649328 | 1.422507024 | 0.87199508 |
| AC053527.2 | 0.976052544 | 0.583642854 | 1.632297153 | 0.926391523 |
| ZNF518A | 0.98985291 | 0.715055517 | 1.370255541 | 0.95098561 |
| SLC12A9-AS1 | 1.00282504 | 0.715049347 | 1.406417705 | 0.986957066 |
