# Supplementary material for: Identification of an immune-related eRNA prognostic signature for clear cell renal cell carcinoma
Source: Aging (Albany NY). 2024 Jan 29;16(3):2232–48. doi: 10.18632/aging.205479 (PMC10911372; doi:10.18632/aging.205479)
Supplement: Supplementary Table 2 [file aging-16-205479-s003.pdf]

SUPPLEMENTARY TABLE

Supplementary Table 2. Prognostic features of IREs and correlation of target genes.

eRNA	KM	Target	cor	cor*P*val
AC003092.1	8.82E-08	TFPI2	0.522450477	4.42E-39
EMX2OS	6.02E-07	EMX2	0.809246934	0
CCDC18-AS1	7.21E-06	CCDC18	0.74912757	0
LINC01389	2.27E-05	FOXD2	0.418684344	2.74E-24
STX4	3.93E-05	FBXL19	0.625144013	0
STX4	3.93E-05	HSD3B7	0.626346923	0
STX4	3.93E-05	ORAI3	0.556775018	0
STX4	3.93E-05	PRSS53	0.650196372	0
SSPO	0.000113124	ZNF467	0.42944313	0
SSPO	0.000113124	ZNF862	0.678603729	0
SLC25A24P1	0.000144688	NBPF4	0.523680588	2.74E-39
SLC25A24P1	0.000144688	NBPF6	0.485813742	2.86E-33
FRY	0.000161361	FRY	1	0
AFG3L1P	0.000331435	FANCA	0.417693895	0
AFG3L1P	0.000331435	MC1R	0.786988388	0
AFG3L1P	0.000331435	SPIRE2	0.424867444	0
AFG3L1P	0.000331435	AFG3L1P	1	0
LINC00671	0.000426575	G6PC	0.703641148	9.92E-82
SPAAR	0.000479272	RECK	0.541849696	1.87E-42
HOTAIR	0.000480838	HOXC11	0.691390398	7.69E-78
HOTAIR	0.000480838	HOXC13	0.411470753	1.94E-23
HOTAIR	0.000480838	HOXC6	0.479131856	2.77E-32
LINC01176	0.00056786	NOD1	0.435177939	2.58E-26
HAGLR	0.001295545	HOXD1	0.855324249	1.74E-155
HAGLR	0.001295545	HOXD3	0.624327104	0
HAGLR	0.001295545	HOXD4	0.729164875	0
HAGLR	0.001295545	HOXD8	0.510689552	0
RASGEF1B	0.001866563	RASGEF1B	1	0
AP001189.3	0.001936226	LRRC32	0.819526937	5.87E-132
RSRP1	0.002838905	RHD	0.448506225	4.93E-28
ZNF337-AS1	0.02853277	NINL	0.521776524	0
ZNF337-AS1	0.02853277	ZNF337	0.607919036	0
